# The educational value of ward rounds as a learning and teaching opportunity for house officers, medical officers, and registrars in Sudanese hospitals: a multi-center cross-sectional study

**DOI:** 10.1186/s12909-023-04404-z

**Published:** 2023-06-08

**Authors:** Mohammed Mahmmoud Fadelallah Eljack, Fadi M. Toum Ahmed, Elfatih A. Hasabo, Mohammed Alfatih, Khabab Abbasher Hussien Mohamed Ahmed, Walaa Elnaiem, Malaz Tarig AbdAlla Mohamed, Alaa Ahmed, Fayha. M. Mdani Hamood, Amina Alfatih Idris Hajhamd, Raga Muawia Mukhtar Ahmed, Alia Fadl Alla Bashir Mansoor, Esraa Mohammed Ahmed, Yageen Makki Elsaid Ahmed Eisa, Nagwa yassin Mohammed Taha, Mohga Elshafie Ahmed, Ammar alemam diab alnour, Duha Osman abdalatheem tayfour, Leenah MS Mohammed, Entisar Abdalla Zin elabdein Ahmed, Samaher Tajeldeen, Maha Ali Abdelrasoul, Iman Magdi Mohamed Balla, Hiba Awadelkareem Osman Fadl, Tawheed Abdelfatah Hamza Ahmed, Abdalla Yousif, Qaswarah A. Abdulrazique, Mohammed Sabri, Hanouf Nasreldeen Dafalla, Mawada fath Alrahaman, Farah Riyad Gafar Mohamed, Alaa Mohammed Osman Basher Ahmed, Noon hatim Khalid Alrabee, Marwa Elbannan Elhassan Mohamed Ali, Monia Mukhtar Ali, Abdelrahim Abdelrazig Ahmed Ibrahim, Ahmed A.Wahaballah, Mohammed Elbagir, Esrar Adel Alkhier, MA Ahmed, Alamin Mustafa, Akram Khalid Altigany, Amna Elaagib, Yahya Almakey, Israa Kamaleldin mohammed Altayeb, Gorashi Humida, Mohamed Hamid Abdelsalam Mohamed, Ahmed Tajalsir Mohamed ali, Omnia Mokhtar Mohammed Ahmed

**Affiliations:** 1 Faculty of Medicine, University of Bakht Alruda, Ad Duwaym, Sudan; 2 Faculty of Medicine, Sinnar University, Sinnar, Sudan; 3 Faculty of Medicine, University of Khartoum, Khartoum, Sudan; 4 Faculty of Medicine, University of Alzaiem Alazhari, Khartoum, Sudan; 5 National University Sudan, Khartoum, Sudan; 6 Faculty of Medicine, University of Bahri, Khartoum, Sudan; 7 Faculty of Medicine, West Kordofan University, Al-Nuhud, Sudan; 8 Faculty of Medicine, Omdurman Alahlia University, Om Durman, Sudan; 9 Faculty of Medicine, Almughtaribeen University, Khartoum, Sudan; 10 Faculty of Medicine, Kordofan University, El Obied, Sudan; 11 Faculty of Medicine, University of Kassala, Kassala, Sudan; 12 Department of Haematology, Faculty of Medical Laboratory Sciences, AL-Neelain University, Khartoum, Sudan; 13 Department of Medical Laboratory, Sudanese Medical Research Association, Khartoum, Sudan; 14 Faculty of Medicine, University of Red Sea, Port Sudan, Sudan; 15 Faculty of Medicine, Omdurman Islamic University, Khartoum, Sudan; 16 Department of Physiology, Omdurman Islamic University, Khartoum, Sudan; 17 Faculty of Medicine, University of Algadarif, Gadarif, Sudan; 18 Faculty of Medicine, Alribat National University, Khartoum, Sudan; 19 Faculty of Medicine, University of Dongola, Dongola, Sudan; 20 Faculty of Medicine, University of ALNeelain, Khartoum, Sudan; 21 Faculty of Medicine, University of Shendi, Shendi, Sudan

**Keywords:** Educational, Training, Teaching, Ward, Round

## Abstract

**Background:**

Ward rounds are a cornerstone in the educational experience of junior doctors and an essential part of teaching patient care. Here, we aimed to assess the doctors’ perception of ward rounds as an educational opportunity and to identify the obstacles faced in conducting a proper ward round in Sudanese hospitals.

**Method:**

A cross-sectional study was conducted from the 15^th^ to the 30^th^ of January 2022 among house officers, medical officers, and registrars in about 50 teaching and referral hospitals in Sudan. House officers and medical officers were considered the learners, while specialist registrars were considered the teachers. Doctors’ perceptions were assessed using an online questionnaire, with a 5-level Likert scale to answer questions.

**Results:**

A total of 2,011 doctors participated in this study (882 house officers, 697 medical officers, and 432 registrars). The participants were aged 26.9 ± 3.2 years, and females constituted about 60% of the sample. An average of 3.1 ± 6.8 ward rounds were conducted per week in our hospitals, with 11.1 ± 20.3 h spent on ward rounds per week.

Most doctors agreed that ward rounds are suitable for teaching patient management (91.3%) and diagnostic investigations (89.1%). Almost all the doctors agreed that being interested in teaching (95.1%) and communicating appropriately with the patients (94.7%) make a good teacher in ward rounds. Furthermore, nearly all the doctors agreed that being interested in learning (94.3%) and communicating appropriately with the teacher (94.5%) make a good student on ward rounds. About 92.8% of the doctors stated that the quality of ward rounds could be improved. The most frequently reported obstacles faced during ward rounds were the noise (70%) and lack of privacy (77%) in the ward environment.

**Conclusion:**

Ward rounds have a special value in teaching patient diagnosis and management. Being interested in teaching/learning and having good communication skills were the two major criteria that make a good teacher/learner. Unfortunately, ward rounds are faced with obstacles related to the ward environment. It is mandatory to ensure the quality of both ward rounds' teaching and environment to optimize the educational value and subsequently improve patient care practice.

**Supplementary Information:**

The online version contains supplementary material available at 10.1186/s12909-023-04404-z.

## Introduction

Ward rounds—sometimes referred to as “walking tightrope”- are a very complex entity that provides a chance for information exchange, demonstration of communication skills, and approach to appropriate clinical decision making [1, 2]. The literature does not provide a single definition of a ward round. Still, it generally refers to medical teams traveling sequentially from inpatient to inpatient and stopping at each to discuss, examine and decide about the details and overall management of care [1, 3]. Topics commonly discussed during rounds include diagnosis, prognosis, and treatment planning [1, 4]. Furthermore, even within one facility the same type of ward round can be undertaken differently [1, 4]. [3, 4]. In a survey conducted by Grant et al. in the late 1980s, senior house officers reported that 58% of their overall learning occurs on rounds [3]. In Sudan, ward rounds have been a cornerstone of medical education in Sudanese medical schools since the establishment of the Faculty of Medicine-University of Khartoum in 1928 (previously Gordon memorial college) and later the University of Gazira in 1976, in addition to lectures, tutorials, and seminars [5]. After graduation, doctors work as under-training doctors (houseman shifts) for a year before taking the license exam to become general practitioners. Once the first part of specialty training is completed, he is known as a specialty registrar, and ward rounds continue to be a more central part of specialty training as well.

Although ward rounds are considered a rich learning opportunity for medical students, and a good strategy, this learning method is prone to multiple problems. The greatest challenges in conducting proper ward rounds include lack of time due to increasing workload, noisy wards, patients not being available, reduced training time, learners at different training levels; and on the other hand, the attending physician is responsible for delivering best patient care practice at the same time [3, 2]. According to B. Roy “With these competing demands, we need a manageable, teachable framework for conducting successful rounds”. Ward-attending physicians currently receive little instruction or guidance on providing innovative and evidence-based instructions to all levels of trainees, exemplifying empathetic, patient-centred communication skills, and delivering high-quality patient care efficiently and cost-effectively, all within a highly complex environment [2].

Another factor that contributes into the complexity of understanding the efficacy of ward-rounds as a teaching method, are factors related to ward environment: such as the complexity of hospital environment, lack of order, large student numbers per health facility and hence, per patient, factors related to the teaching staff: such as their training to conduct ward rounds, and their preparedness for the ward rounds themselves. And factors related to students, such as: their preparedness for the ward rounds and their interaction. All these factors contribute to a complex healthcare setting that can affect the quality and efficacy of ward rounds and their benefit to students. (Unique problem).

In recent years, new educational strategies have been implemented, such as clinical skills lab, sessions facilitated by small groups, and problem-based learning sessions. However, there is still is a necessity to put effort into improving the quality and the educational value of ward rounds to achieve the highest benefit from this cornerstone learning method. In Sudan, the process of improving ward rounds is hampered by a lack of original research studies in literature, as well as the fact that literature on this topic addresses ward rounds that were conducted in different settings, such as different types of wards, different hospitals, different countries and further, different levels of training including teachers [1, 3]. Factors that contribute even more to the ambiguity of the situation is Sudan's poor healthcare settings, large number of patients per ward, and the large number of students attending ward round sessions per doctor, as most universities in Sudan suffer from a lack in teaching staff due to doctors’ immigration. Along with the fact that Sudan has returned to (UTC + 2) Time zone on the 1^st^ November 2017 which has an impact on reducing the time into which both patient care services and training conducted.

Hence, drawing on the premises above, and the insufficient literature that attends to this problem in Sudan, this study aims to assess the quality and efficacy of ward rounds as a learning method and educational opportunity and to identify the obstacles to conducting a proper ward round in Sudanese hospitals. It represents the first of its type in Africa and the Middle-East. It also provides an overview of successful rounds’ structural components and common difficulties for attending Sudanese physicians who want to improve their performance.

## Methodology

### Study setting

A cross-sectional analytic study was conducted between the 15th and 30th of January 2022, focusing on house officers, medical doctors, and registrars in Sudanese teaching and referral hospitals. The study included approximately 50 hospitals, 26 of which were located in Khartoum, the capital of Khartoum state, and 24 hospitals in Sudan’s other 17 states. In this study, house officers and medical officers were regarded as learners, while medical specialist registrars were regarded as teachers.

Doctors working in hospitals and not registered as trainees by Sudan Medical Council (SMC) were excluded from the study.

## Sampling

### Sampling technique

A conventional non-probability random sampling was done for all house officers, medical officers, and registrars within Sudanese teaching and referral hospitals between 15^th^ January 2022 to 30^th^ January 2022.

## Data collection tool and techniques

A well-structured self-administered questionnaire was distributed by a team of more than 40 doctors to collect data in both Google forms (distributed through social media including Facebook, WhatsApp, LinkedIn…etc.) and hard copies to all participants. A minimum sample size of 288 was estimated using the online Rao soft sample size calculator, using a 95% confidence interval, 50% response distribution, 5% margin of error, and an estimated population size of 15,000. This was done after distributing the form to 30 participants first, and recording their responses and feedback to see how each participant interpreted the question. Their feedback, if any, was considered when making changes to the questionnaire based on this pilot experiment. The questionnaire used in this study can be found as [Additional file 1].

Before beginning the process, data collectors were given detailed explanations for each question. An introduction page contains information about the principal investigators’ identities, links to their official profiles, contact information, and the purpose of the study and its significance to the scientific community. To ensure anonymity and confidentiality, participants’ IP addresses were not collected, and only the principal investigator had access to the survey account. The survey is expected to take 3 to 5 min to complete. Questions were answered using a five-level Likert scale, with free text for descriptive answers for some variables.

The questionnaire contained the following domains: socio-demographic data, the educational value of current ward rounds, assessment of ward rounds as a learning and teaching opportunity, obstacles to learning and teaching ward rounds, the effect of the ward rounds structure on learning and teaching, and the effect of the teacher and learner on the educational opportunities of ward rounds.

## Data management and statistical analysis

Data was manually entered, cleaned, and analyzed using the R software version 4.0.2. The results were presented in figures and tables as numbers (percentages) and mean (Standard deviation; SD). *P*-value < 0.05, as well as a 95% confidence interval (CI) not including the null value, was considered statistically significant. The Chi-square test or Fisher exact test was used to find if there was a significant difference between the groups.

## Results

The overall response rate was 67% (2,011/3,000). A total of 2,011 doctors participated in this study; 882 (43.9%) were house officers, 697 (34.7%) were medical officers, and 432 (21.5%) were registrars. The participants had a mean age of 26.9 ± 3.2 years, and females constituted about 60% of the sample. The majority of the participants were from the Department of Medicine (27.2%), followed by Paediatrics (21.7%), Surgery (19.2%), and Obstetrics and Gynaecology (16.5%). The overall number of rounds conducted per week was 3.1 ± 6.8, with a total of 11.1 ± 20.3 weekly hours. Each unit contained an average of 3.6 ± 3.8 registrars and 6.8 ± 7.5 house officers. Characteristics of the study participants were summarized in Table 1.

**Table 1:** Characteristics of the study participants

Variables	N	Overall *N* = 2,011^a^	Position			*p*-value^b^
			House officer *N* = 882^a^	Medical officer *N* = 697^a^	Registrar *N* = 432^a^	
Age, years	1,961	26.9 ± 3.2	25.3 ± 1.8	27.0 ± 2.8	29.9 ± 3.6	
Gender	2,006					0.067
Female		1,208 (60.2%)	517 (58.7%)	410 (59.2%)	281 (65.0%)	
Male		798 (39.8%)	364 (41.3%)	283 (40.8%)	151 (35.0%)	
Department	2,006					< 0.001
Anesthesia		6 (0.3%)	0 (0.0%)	0 (0.0%)	6 (1.4%)	
Cardiology		1 (0.0%)	0 (0.0%)	1 (0.1%)	0 (0.0%)	
Chest medicine		3 (0.1%)	0 (0.0%)	0 (0.0%)	3 (0.7%)	
Clinical Immunology		3 (0.1%)	0 (0.0%)	0 (0.0%)	3 (0.7%)	
Critical care		30 (1.5%)	0 (0.0%)	28 (4.0%)	2 (0.5%)	
Pathology		3 (0.15%)	0 (0.0%)	0 (0.0%)	3 (0.69%)	
Dermatology		21 (1.0%)	1 (0.1%)	3 (0.4%)	17 (3.9%)	
Emergency medicine		90 (4.5%)	2 (0.2%)	76 (11.0%)	12 (2.8%)	
ENT		17 (0.8%)	0 (0.0%)	14 (2.0%)	3 (0.7%)	
Family medicine		7 (0.3%)	0 (0.0%)	2 (0.3%)	5 (1.2%)	
Hematology		1 (0.0%)	0 (0.0%)	0 (0.0%)	1 (0.2%)	
Covid-19 isolation		6 (0.3%)	0 (0.0%)	6 (0.9%)	0 (0.0%)	
Medicine		546 (27.2%)	246 (27.9%)	200 (28.9%)	100 (23.1%)	
Neuro medicine		1 (0.0%)	0 (0.0%)	0 (0.0%)	1 (0.2%)	
Neurosurgery		12 (0.6%)	0 (0.0%)	9 (1.3%)	3 (0.7%)	
Obs/Gyne		331 (16.5%)	211 (24.0%)	37 (5.3%)	83 (19.2%)	
Oncology		1 (0.0%)	0 (0.0%)	0 (0.0%)	1 (0.2%)	
Ophthalmology		9 (0.4%)	0 (0.0%)	6 (0.9%)	3 (0.7%)	
Orthopedic surgery and trauma		24 (1.2%)	2 (0.2%)	15 (2.2%)	7 (1.6%)	
Pathology		7 (0.3%)	0 (0.0%)	1 (0.1%)	6 (1.4%)	
Pediatric		435 (21.7%)	247 (28.0%)	120 (17.3%)	68 (15.7%)	
Pediatric surgery		10 (0.5%)	1 (0.1%)	4 (0.6%)	5 (1.2%)	
Plastic surgery		2 (0.1%)	0 (0.0%)	2 (0.3%)	0 (0.0%)	
Psychiatry		35 (1.7%)	0 (0.0%)	7 (1.0%)	27 (6.5%)	
Radiology		7 (0.3%)	0 (0.0%)	2 (0.3%)	5 (1.2%)	
Nephrology		2 (0.1%)	0 (0.0%)	2 (0.3%)	0 (0.0%)	
Surgery		385 (19.2%)	171 (19.4%)	155 (22.4%)	59 (13.7%)	
Urology		11 (0.5%)	0 (0.0%)	3 (0.4%)	8 (1.9%)	
Years from graduation	2,002	2.8 ± 2.5	1.2 ± 0.8	3.2 ± 1.7	5.6 ± 3.0	
The number of ward rounds conducted per week	2,005	3.1 ± 6.8	3.2 ± 5.9	3.3 ± 9.3	2.4 ± 1.9	
Total hours spent on word rounds per week	2,002	11.1 ± 20.3	12.1 ± 17.5	11.0 ± 16.4	9.4 ± 29.4	
The number of house officers/medical officers in your unite	1,999	6.8 ± 7.5	7.2 ± 6.4	6.7 ± 6.4	6.0 ± 10.6	
The number of registrars in your unite	2,001	3.6 ± 3.8	3.1 ± 2.6	2.7 ± 2.7	6.0 ± 5.9	

^a^Data were presented as Mean ± SD and n (%)

^b^Pearson's Chi-squared test or independent T-test

A large majority of the doctors agreed that ward rounds are suitable for teaching patient management (91.3%), diagnostic investigations (89.1%), history taking (88.3%), physical examination (87%), communication skills (85.9%), time management skills (73.7%), and record keeping (72.3%). A fewer percentage of the doctors agreed that ward rounds are suitable for teaching basic sciences (56.4%). A summary of the doctors' perception of the educational value of ward rounds is available in Table 2.

**Table 2:** Summary of the doctors' perception of the educational value of ward rounds

Variables	N	Overall *N* = 2,011^a^	Position		
			House officer *N* = 882^a^	Medical officer *N* = 697^a^	Registrar *N* = 432^a^
Ward rounds have been a good opportunity to teach (learn):					
History Taking	2,006				
Strongly agree		892 (44.5%)	332 (37.7%)	334 (48.2%)	226 (52.3%)
Agree		879 (43.8%)	417 (47.3%)	297 (42.9%)	165 (38.2%)
Neither agree nor disagree		116 (5.8%)	66 (7.5%)	33 (4.8%)	17 (3.9%)
Disagree		87 (4.3%)	53 (6.0%)	20 (2.9%)	14 (3.2%)
Strongly disagree		32 (1.6%)	13 (1.5%)	9 (1.3%)	10 (2.3%)
Physical examination	2,006				
Strongly agree		942 (47.0%)	341 (38.7%)	367 (53.0%)	234 (54.2%)
Agree		803 (40.0%)	394 (44.7%)	256 (36.9%)	153 (35.4%)
Neither agree nor disagree		151 (7.5%)	81 (9.2%)	43 (6.2%)	27 (6.2%)
Disagree		94 (4.7%)	56 (6.4%)	25 (3.6%)	13 (3.0%)
Strongly disagree		16 (0.8%)	9 (1.0%)	2 (0.3%)	5 (1.2%)
Diagnostic investigations	2,006				
Strongly agree		827 (41.2%)	333 (37.8%)	294 (42.4%)	200 (46.3%)
Agree		961 (47.9%)	432 (49.0%)	336 (48.5%)	193 (44.7%)
Neither agree nor disagree		132 (6.6%)	63 (7.2%)	39 (5.6%)	30 (6.9%)
Disagree		66 (3.3%)	39 (4.4%)	22 (3.2%)	5 (1.2%)
Strongly disagree		20 (1.0%)	14 (1.6%)	2 (0.3%)	4 (0.9%)
Patient management	2,006				
Strongly agree		950 (47.4%)	372 (42.2%)	345 (49.8%)	233 (53.9%)
Agree		880 (43.9%)	406 (46.1%)	299 (43.1%)	175 (40.5%)
Neither agree nor disagree		108 (5.4%)	65 (7.4%)	34 (4.9%)	9 (2.1%)
Disagree		52 (2.6%)	27 (3.1%)	13 (1.9%)	12 (2.8%)
Strongly disagree		16 (0.8%)	11 (1.2%)	2 (0.3%)	3 (0.7%)
Communication skills	2,006				
Strongly agree		881 (43.9%)	341 (38.7%)	318 (45.9%)	222 (51.4%)
Agree		843 (42.0%)	386 (43.8%)	288 (41.6%)	169 (39.1%)
Neither agree nor disagree		171 (8.5%)	99 (11.2%)	53 (7.6%)	19 (4.4%)
Disagree		87 (4.3%)	46 (5.2%)	25 (3.6%)	16 (3.7%)
Strongly disagree		24 (1.2%)	9 (1.0%)	9 (1.3%)	6 (1.4%)
Time Management Skills	2,006				
Strongly agree		638 (31.8%)	242 (27.5%)	230 (33.2%)	166 (38.4%)
Agree		830 (41.4%)	366 (41.5%)	287 (41.4%)	177 (41.0%)
Neither agree nor disagree		269 (13.4%)	139 (15.8%)	93 (13.4%)	37 (8.6%)
Disagree		234 (11.7%)	121 (13.7%)	72 (10.4%)	41 (9.5%)
Strongly disagree		35 (1.7%)	13 (1.5%)	11 (1.6%)	11 (2.5%)
Record Keeping	2,006				
Strongly agree		510 (25.4%)	183 (20.8%)	190 (27.4%)	137 (31.7%)
Agree		941 (46.9%)	417 (47.3%)	314 (45.3%)	210 (48.6%)
Neither agree nor disagree		348 (17.3%)	182 (20.7%)	116 (16.7%)	50 (11.6%)
Disagree		177 (8.8%)	89 (10.1%)	62 (8.9%)	26 (6.0%)
Strongly disagree		30 (1.5%)	10 (1.1%)	11 (1.6%)	9 (2.1%)
Basic Sciences	2,006				
Strongly agree		372 (18.5%)	135 (15.3%)	146 (21.1%)	91 (21.1%)
Agree		760 (37.9%)	327 (37.1%)	262 (37.8%)	171 (39.6%)
Neither agree nor disagree		380 (18.9%)	175 (19.9%)	125 (18.0%)	80 (18.5%)
Disagree		400 (19.9%)	192 (21.8%)	133 (19.2%)	75 (17.4%)
Strongly disagree		94 (4.7%)	52 (5.9%)	27 (3.9%)	15 (3.5%)

^a^Data were presented as n (%)

A high percentage of the study participants (92.8%) agreed that ward rounds could be made into a better learning experience. The reported obstacles faced on ward rounds were the lack of privacy (77%) and noise (70%) in the ward environment. Moreover, over half of the participants reported a lack of nurses (65%), a large number of patients (63.5%), changes in team structure (62.5%), lack of time (58.6%), and patient complaints (58.4%) as challenging obstacles. The patients' meal time (42.5%) or unavailability (45.7%) and not knowing the patients (37.6%) were only reported by a minority of the doctors as obstacles. The doctors’ perception of the obstacles faced on ward rounds was summarized in Table 3.

**Table 3:** Summary of the doctors' perception of the obstacles faced on ward rounds

Variables	N	Overall *N* = 2,011^a^	Position		
			House officer *N* = 882^a^	Medical officer *N* = 697^a^	Registrar *N* = 432^a^
Do you agree that ward rounds could be made into a better learning experience?	2,006				
Strongly agree		1,087 (54.2%)	448 (50.9%)	377 (54.4%)	262 (60.6%)
Agree		774 (38.6%)	358 (40.6%)	266 (38.4%)	150 (34.7%)
Neither agree nor disagree		71 (3.5%)	37 (4.2%)	26 (3.8%)	8 (1.9%)
Disagree		57 (2.8%)	27 (3.1%)	22 (3.2%)	8 (1.9%)
Strongly disagree		17 (0.8%)	11 (1.2%)	2 (0.3%)	4 (0.9%)
Obstacles to learning (teaching) on ward rounds:					
Lack of time	2,006				
Strongly agree		321 (16.0%)	120 (13.6%)	118 (17.0%)	83 (19.2%)
Agree		855 (42.6%)	372 (42.2%)	296 (42.7%)	187 (43.3%)
Neither agree nor disagree		380 (18.9%)	181 (20.5%)	133 (19.2%)	66 (15.3%)
Disagree		398 (19.8%)	185 (21.0%)	134 (19.3%)	79 (18.3%)
Strongly disagree		52 (2.6%)	23 (2.6%)	12 (1.7%)	17 (3.9%)
Number of patients	2,006				
Strongly agree		343 (17.1%)	149 (16.9%)	120 (17.3%)	74 (17.1%)
Agree		931 (46.4%)	412 (46.8%)	326 (47.0%)	193 (44.7%)
Neither agree nor disagree		312 (15.6%)	150 (17.0%)	110 (15.9%)	52 (12.0%)
Disagree		380 (18.9%)	152 (17.3%)	128 (18.5%)	100 (23.1%)
Strongly disagree		40 (2.0%)	18 (2.0%)	9 (1.3%)	13 (3.0%)
Team structure changes too often	2,006				
Strongly agree		313 (15.6%)	131 (14.9%)	118 (17.0%)	64 (14.8%)
Agree		940 (46.9%)	397 (45.1%)	345 (49.8%)	198 (45.8%)
Neither agree nor disagree		431 (21.5%)	206 (23.4%)	138 (19.9%)	87 (20.1%)
Disagree		299 (14.9%)	138 (15.7%)	88 (12.7%)	73 (16.9%)
Strongly disagree		23 (1.1%)	9 (1.0%)	4 (0.6%)	10 (2.3%)
I don’t know the patients	2,006				
Strongly agree		174 (8.7%)	67 (7.6%)	68 (9.8%)	39 (9.0%)
Agree		580 (28.9%)	266 (30.2%)	203 (29.3%)	111 (25.7%)
Neither agree nor disagree		394 (19.6%)	189 (21.5%)	125 (18.0%)	80 (18.5%)
Disagree		741 (36.9%)	305 (34.6%)	266 (38.4%)	170 (39.4%)
Strongly disagree		117 (5.8%)	54 (6.1%)	31 (4.5%)	32 (7.4%)
Ward's environment was too noisy	2,006				
Strongly agree		534 (26.6%)	221 (25.1%)	199 (28.7%)	114 (26.4%)
Agree		870 (43.4%)	378 (42.9%)	300 (43.3%)	192 (44.4%)
Neither agree nor disagree		236 (11.8%)	117 (13.3%)	77 (11.1%)	42 (9.7%)
Disagree		314 (15.7%)	141 (16.0%)	100 (14.4%)	73 (16.9%)
Strongly disagree		52 (2.6%)	24 (2.7%)	17 (2.5%)	11 (2.5%)
Ward environment lacks privacy	2,006				
Strongly agree		673 (33.5%)	266 (30.2%)	247 (35.6%)	160 (37.0%)
Agree		872 (43.5%)	394 (44.7%)	295 (42.6%)	183 (42.4%)
Neither agree nor disagree		219 (10.9%)	108 (12.3%)	75 (10.8%)	36 (8.3%)
Disagree		213 (10.6%)	98 (11.1%)	67 (9.7%)	48 (11.1%)
Strongly disagree		29 (1.4%)	15 (1.7%)	9 (1.3%)	5 (1.2%)
The Ward environment lacks nursing staff	2,006				
Strongly agree		503 (25.1%)	225 (25.5%)	172 (24.8%)	106 (24.5%)
Agree		803 (40.0%)	346 (39.3%)	265 (38.2%)	192 (44.4%)
Neither agree nor disagree		272 (13.6%)	121 (13.7%)	94 (13.6%)	57 (13.2%)
Disagree		373 (18.6%)	156 (17.7%)	144 (20.8%)	73 (16.9%)
Strongly disagree		55 (2.7%)	33 (3.7%)	18 (2.6%)	4 (0.9%)
Patient complaint	2,006				
Strongly agree		289 (14.4%)	124 (14.1%)	106 (15.3%)	59 (13.7%)
Agree		882 (44.0%)	381 (43.2%)	322 (46.5%)	179 (41.4%)
Neither agree nor disagree		406 (20.2%)	194 (22.0%)	125 (18.0%)	87 (20.1%)
Disagree		380 (18.9%)	162 (18.4%)	128 (18.5%)	90 (20.8%)
Strongly disagree		49 (2.4%)	20 (2.3%)	12 (1.7%)	17 (3.9%)
Patient meal time	2,006				
Strongly agree		203 (10.1%)	92 (10.4%)	69 (10.0%)	42 (9.7%)
Agree		650 (32.4%)	261 (29.6%)	236 (34.1%)	153 (35.4%)
Neither agree nor disagree		491 (24.5%)	230 (26.1%)	172 (24.8%)	89 (20.6%)
Disagree		586 (29.2%)	256 (29.1%)	201 (29.0%)	129 (29.9%)
Strongly disagree		76 (3.8%)	42 (4.8%)	15 (2.2%)	19 (4.4%)
Patient not available	2,006				
Strongly agree		223 (11.1%)	95 (10.8%)	79 (11.4%)	49 (11.3%)
Agree		695 (34.6%)	307 (34.8%)	246 (35.5%)	142 (32.9%)
Neither agree nor disagree		406 (20.2%)	185 (21.0%)	141 (20.3%)	80 (18.5%)
Disagree		587 (29.3%)	250 (28.4%)	203 (29.3%)	134 (31.0%)
Strongly disagree		95 (4.7%)	44 (5.0%)	24 (3.5%)	27 (6.2%)

^a^Data were presented as n (%)

The participants were asked about the characteristics that make a good teacher in ward rounds. Nearly all the participants agreed on being interested in teaching (95.1%), communicating appropriately with the patients (94.7%), understanding the learning needs (94.4%), communicating appropriately with the students (93.4%), providing feedback (93.1%), and approachability (91.6%). Additionally, most of the participants agreed on teaching patiently (87.5%), teaching in the presence of registrars (87.4%), taking time to explain (85.2%), slow steady teaching with interest (85%), being respected by the learners (82.4%), choosing interesting topics (79.6%), and having experience in teaching medical students (71.4%). Teaching in the presence of many registrars, avoiding intimidating manners, and being known by the students were chosen by a fewer percentage of the doctors (69.5%, 67.9%, and 56.2%, respectively). A summary of the doctors' perception of the characteristics that make a good teacher in ward rounds is available in Table 4.

**Table 4:** Summary of the doctors’ perception about the characteristics that make a good teacher in ward rounds

Variables	N	Overall *N* = 2,011^a^	Position		
			House officer *N* = 882^a^	Medical officer *N* = 697^a^	Registrar *N* = 432^a^
What makes a good teacher in ward rounds:					
Someone you know	2,006				
Strongly agree		268 (13.4%)	112 (12.7%)	95 (13.7%)	61 (14.1%)
Agree		858 (42.8%)	390 (44.3%)	290 (41.8%)	178 (41.2%)
Neither agree nor disagree		515 (25.7%)	225 (25.5%)	194 (28.0%)	96 (22.2%)
Disagree		322 (16.1%)	134 (15.2%)	104 (15.0%)	84 (19.4%)
Strongly disagree		43 (2.1%)	20 (2.3%)	10 (1.4%)	13 (3.0%)
Someone you respect	2,006				
Strongly agree		525 (26.2%)	224 (25.4%)	185 (26.7%)	116 (26.9%)
Agree		1,128 (56.2%)	487 (55.3%)	394 (56.9%)	247 (57.2%)
Neither agree nor disagree		246 (12.3%)	123 (14.0%)	81 (11.7%)	42 (9.7%)
Disagree		97 (4.8%)	44 (5.0%)	30 (4.3%)	23 (5.3%)
Strongly disagree		10 (0.5%)	3 (0.3%)	3 (0.4%)	4 (0.9%)
Presence of Many consultants in round	2,006				
Strongly agree		573 (28.6%)	234 (26.6%)	212 (30.6%)	127 (29.4%)
Agree		821 (40.9%)	358 (40.6%)	277 (40.0%)	186 (43.1%)
Neither agree nor disagree		313 (15.6%)	151 (17.1%)	106 (15.3%)	56 (13.0%)
Disagree		265 (13.2%)	123 (14.0%)	85 (12.3%)	57 (13.2%)
Strongly disagree		34 (1.7%)	15 (1.7%)	13 (1.9%)	6 (1.4%)
Presence of registrars	2,006				
Strongly agree		721 (35.9%)	342 (38.8%)	235 (33.9%)	144 (33.3%)
Agree		1,034 (51.5%)	430 (48.8%)	365 (52.7%)	239 (55.3%)
Neither agree nor disagree		162 (8.1%)	74 (8.4%)	65 (9.4%)	23 (5.3%)
Disagree		74 (3.7%)	30 (3.4%)	22 (3.2%)	22 (5.1%)
Strongly disagree		15 (0.7%)	5 (0.6%)	6 (0.9%)	4 (0.9%)
Interest towards teaching	2,006				
Strongly agree		1,087 (54.2%)	459 (52.1%)	389 (56.1%)	239 (55.3%)
Agree		821 (40.9%)	365 (41.4%)	275 (39.7%)	181 (41.9%)
Neither agree nor disagree		76 (3.8%)	45 (5.1%)	22 (3.2%)	9 (2.1%)
Disagree		19 (0.9%)	10 (1.1%)	7 (1.0%)	2 (0.5%)
Strongly disagree		3 (0.1%)	2 (0.2%)	0 (0.0%)	1 (0.2%)
Someone who is not intimidating	2,006				
Strongly agree		477 (23.8%)	219 (24.9%)	154 (22.2%)	104 (24.1%)
Agree		884 (44.1%)	388 (44.0%)	316 (45.6%)	180 (41.7%)
Neither agree nor disagree		493 (24.6%)	214 (24.3%)	167 (24.1%)	112 (25.9%)
Disagree		131 (6.5%)	51 (5.8%)	49 (7.1%)	31 (7.2%)
Strongly disagree		21 (1.0%)	9 (1.0%)	7 (1.0%)	5 (1.2%)
Someone who is not in a hurry	2,006				
Strongly agree		782 (39.0%)	340 (38.6%)	264 (38.1%)	178 (41.2%)
Agree		972 (48.5%)	425 (48.2%)	332 (47.9%)	215 (49.8%)
Neither agree nor disagree		168 (8.4%)	85 (9.6%)	62 (8.9%)	21 (4.9%)
Disagree		77 (3.8%)	30 (3.4%)	31 (4.5%)	16 (3.7%)
Strongly disagree		7 (0.3%)	1 (0.1%)	4 (0.6%)	2 (0.5%)
Someone who can communicate with you	2,006				
Strongly agree		999 (49.8%)	435 (49.4%)	349 (50.4%)	215 (49.8%)
Agree		874 (43.6%)	379 (43.0%)	307 (44.3%)	188 (43.5%)
Neither agree nor disagree		104 (5.2%)	56 (6.4%)	28 (4.0%)	20 (4.6%)
Disagree		22 (1.1%)	10 (1.1%)	6 (0.9%)	6 (1.4%)
Strongly disagree		7 (0.3%)	1 (0.1%)	3 (0.4%)	3 (0.7%)
Someone who can communicate with the patient	2,006				
Strongly agree		1,030 (51.3%)	432 (49.0%)	362 (52.2%)	236 (54.6%)
Agree		870 (43.4%)	394 (44.7%)	299 (43.1%)	177 (41.0%)
Neither agree nor disagree		76 (3.8%)	44 (5.0%)	20 (2.9%)	12 (2.8%)
Disagree		24 (1.2%)	11 (1.2%)	9 (1.3%)	4 (0.9%)
Strongly disagree		6 (0.3%)	0 (0.0%)	3 (0.4%)	3 (0.7%)
Someone who can provide feedback	2,006				
Strongly agree		910 (45.4%)	395 (44.8%)	321 (46.3%)	194 (44.9%)
Agree		956 (47.7%)	416 (47.2%)	336 (48.5%)	204 (47.2%)
Neither agree nor disagree		115 (5.7%)	54 (6.1%)	31 (4.5%)	30 (6.9%)
Disagree		23 (1.1%)	16 (1.8%)	5 (0.7%)	2 (0.5%)
Strongly disagree		2 (0.1%)	0 (0.0%)	0 (0.0%)	2 (0.5%)
Approachable	2,006				
Strongly agree		849 (42.3%)	370 (42.0%)	287 (41.4%)	192 (44.4%)
Agree		989 (49.3%)	428 (48.6%)	356 (51.4%)	205 (47.5%)
Neither agree nor disagree		146 (7.3%)	71 (8.1%)	44 (6.3%)	31 (7.2%)
Disagree		19 (0.9%)	10 (1.1%)	6 (0.9%)	3 (0.7%)
Strongly disagree		3 (0.1%)	2 (0.2%)	0 (0.0%)	1 (0.2%)
Someone who takes time to explain	2,006				
Strongly agree		859 (42.8%)	374 (42.5%)	293 (42.3%)	192 (44.4%)
Agree		850 (42.4%)	377 (42.8%)	293 (42.3%)	180 (41.7%)
Neither agree nor disagree		175 (8.7%)	78 (8.9%)	60 (8.7%)	37 (8.6%)
Disagree		107 (5.3%)	44 (5.0%)	44 (6.3%)	19 (4.4%)
Strongly disagree		15 (0.7%)	8 (0.9%)	3 (0.4%)	4 (0.9%)
The topic he chose	2,006				
Strongly agree		601 (30.0%)	267 (30.3%)	201 (29.0%)	133 (30.8%)
Agree		995 (49.6%)	433 (49.1%)	355 (51.2%)	207 (47.9%)
Neither agree nor disagree		289 (14.4%)	133 (15.1%)	93 (13.4%)	63 (14.6%)
Disagree		114 (5.7%)	46 (5.2%)	44 (6.3%)	24 (5.6%)
Strongly disagree		7 (0.3%)	2 (0.2%)	0 (0.0%)	5 (1.2%)
Seem to interact more with medical students	2,006				
Strongly agree		509 (25.4%)	252 (28.6%)	157 (22.7%)	100 (23.1%)
Agree		922 (46.0%)	379 (43.0%)	339 (48.9%)	204 (47.2%)
Neither agree nor disagree		390 (19.4%)	167 (19.0%)	139 (20.1%)	84 (19.4%)
Disagree		159 (7.9%)	71 (8.1%)	51 (7.4%)	37 (8.6%)
Strongly disagree		26 (1.3%)	12 (1.4%)	7 (1.0%)	7 (1.6%)
Slow steady ward rounds with interested doctors are the best	2,006				
Strongly agree		804 (40.1%)	359 (40.7%)	273 (39.4%)	172 (39.8%)
Agree		900 (44.9%)	394 (44.7%)	309 (44.6%)	197 (45.6%)
Neither agree nor disagree		193 (9.6%)	84 (9.5%)	72 (10.4%)	37 (8.6%)
Disagree		99 (4.9%)	40 (4.5%)	38 (5.5%)	21 (4.9%)
Strongly disagree		10 (0.5%)	4 (0.5%)	1 (0.1%)	5 (1.2%)
Someone who understands the learning needs	2,006				
Strongly agree		970 (48.4%)	427 (48.5%)	331 (47.8%)	212 (49.1%)
Agree		922 (46.0%)	397 (45.1%)	329 (47.5%)	196 (45.4%)
Neither agree nor disagree		95 (4.7%)	51 (5.8%)	26 (3.8%)	18 (4.2%)
Disagree		14 (0.7%)	6 (0.7%)	6 (0.9%)	2 (0.5%)
Strongly disagree		5 (0.2%)	0 (0.0%)	1 (0.1%)	4 (0.9%)

^a^Data were presented as n (%)

Furthermore, the doctors were asked about their perception of what makes a good learner in ward rounds. Almost all the participants agreed on communicating appropriately with the teacher (94.5%), being interested in learning (94.3%), and communicating appropriately with the patients (93.9%). The majority agreed on having good knowledge (87.9%) and not being in a hurry (87.2%). Moreover, about half of the doctors agreed on being known by the teacher (53%). The doctors' perception of what makes a good learner in ward rounds is summarized in Table 5.

**Table 5:** Summary of the doctors' perception of the characteristics that make a good learner in ward rounds

Variables	N	Overall *N* = 2,011^a^	Position		
			House officer *N* = 882^a^	Medical officer *N* = 697^a^	Registrar *N* = 432^a^
What makes a good student on a ward round:					
Someone you know	2,006				
Strongly agree		321 (16.0%)	140 (15.9%)	123 (17.7%)	58 (13.4%)
Agree		743 (37.0%)	357 (40.5%)	235 (33.9%)	151 (35.0%)
Neither agree nor disagree		591 (29.5%)	244 (27.7%)	211 (30.4%)	136 (31.5%)
Disagree		307 (15.3%)	117 (13.3%)	113 (16.3%)	77 (17.8%)
Strongly disagree		44 (2.2%)	23 (2.6%)	11 (1.6%)	10 (2.3%)
Interest towards learning	2,006				
Strongly agree		957 (47.7%)	397 (45.1%)	348 (50.2%)	212 (49.1%)
Agree		934 (46.6%)	419 (47.6%)	310 (44.7%)	205 (47.5%)
Neither agree nor disagree		88 (4.4%)	50 (5.7%)	27 (3.9%)	11 (2.5%)
Disagree		21 (1.0%)	11 (1.2%)	8 (1.2%)	2 (0.5%)
Strongly disagree		6 (0.3%)	4 (0.5%)	0 (0.0%)	2 (0.5%)
Good level of knowledge	2,006				
Strongly agree		776 (38.7%)	347 (39.4%)	268 (38.7%)	161 (37.3%)
Agree		987 (49.2%)	419 (47.6%)	345 (49.8%)	223 (51.6%)
Neither agree nor disagree		166 (8.3%)	76 (8.6%)	57 (8.2%)	33 (7.6%)
Disagree		68 (3.4%)	34 (3.9%)	21 (3.0%)	13 (3.0%)
Strongly disagree		9 (0.4%)	5 (0.6%)	2 (0.3%)	2 (0.5%)
Someone who is not in a hurry	2,006				
Strongly agree		733 (36.5%)	322 (36.5%)	244 (35.2%)	167 (38.7%)
Agree		1,017 (50.7%)	446 (50.6%)	357 (51.5%)	214 (49.5%)
Neither agree nor disagree		188 (9.4%)	92 (10.4%)	62 (8.9%)	34 (7.9%)
Disagree		57 (2.8%)	16 (1.8%)	27 (3.9%)	14 (3.2%)
Strongly disagree		11 (0.5%)	5 (0.6%)	3 (0.4%)	3 (0.7%)
Someone who can communicate with you	2,006				
Strongly agree		815 (40.6%)	366 (41.5%)	276 (39.8%)	173 (40.0%)
Agree		1,021 (50.9%)	434 (49.3%)	360 (51.9%)	227 (52.5%)
Neither agree nor disagree		141 (7.0%)	68 (7.7%)	48 (6.9%)	25 (5.8%)
Disagree		25 (1.2%)	11 (1.2%)	9 (1.3%)	5 (1.2%)
Strongly disagree		4 (0.2%)	2 (0.2%)	0 (0.0%)	2 (0.5%)
Someone who can communicate with patients	2,006				
Strongly agree		940 (46.9%)	406 (46.1%)	329 (47.5%)	205 (47.5%)
Agree		943 (47.0%)	417 (47.3%)	321 (46.3%)	205 (47.5%)
Neither agree nor disagree		92 (4.6%)	48 (5.4%)	33 (4.8%)	11 (2.5%)
Disagree		18 (0.9%)	6 (0.7%)	5 (0.7%)	7 (1.6%)
Strongly disagree		13 (0.6%)	4 (0.5%)	5 (0.7%)	4 (0.9%)

^a^Data were presented as n (%)

## Discussion

The purpose of this study was to evaluate the effectiveness and quality of the ward round as a teaching tool for house officers, medical officers, and registrars in Sudan, while also identifying the barriers to a good ward round.

2,011 doctors made up the study's sample, which is the highest sample size among research with similar goals that we are aware of [1–12]. 34.7% (*n* = 697) of the participants were medical officers, 21.5% (*n* = 432) were registrars, and 43.9% (*n* = 882) were house officers, this could be attributed to the fact that the number of house officers and medical officers is higher than registrars in hospitals as they are the base of the pyramid in the hospital. The participants' average age was 26 years + \- 3 years, and 60% of them were female. Participants in a study by Claridge [3] were 25 years old on average, with a 5-year standard deviation. The mean age in other research was 27 years [4]. Compared to registrars, house officers and medical officers are more represented in this study. This was evident in our study as in other literature [1–4]. Also, the larger proportion of female participants was comparable to other studies [1, 3].

Regarding specialties, in some publications, the representation of medical specialties was higher than that of surgical specialties [1, 4], while in other papers, house officers were the only group represented [3] or medical specialty trainees were the only specialty investigated [2, 4]. The majority of residents in this study (27.2%) were from the Department of Medicine, which was followed by the Departments of Paediatrics (21.7%), Surgery (19.2%), and Obstetrics and Gynaecology (16.5%). Approximately 3 to 7 rounds were conducted each week altogether. The average was 5 rounds per week with a total of 11.1 to 20.3 weekly hours, which is greater than the average 4 rounds per week recorded in the literature [1, 4]. With a mean of 6.5 rounds per week [3]. The range of weekly hours in the literature varied from 10.5 h [3], 11 h [1], and 13 h [4]. In this study, there were approximately 7 to 8 house officers and approximately 4 registrars in each medical unit. Table 1 summarizes the characteristics of the study participants.

When asked which training domains ward rounds serve a good opportunity to teach, respondents reported that ward rounds are appropriate for teaching patient management (91.3%), diagnostic investigations (89.1%), history taking (88.3%), physical examination (87%), communication skills (85.9%), time management skills (73.7%), and record keeping (72.3%), according to the vast majority of doctors. Only (56.4%) agreed that teaching basic sciences may be done during ward rounds. Table 2 provides a summary of the doctors' opinions on the educational value of ward rounds. These findings are perfectly consistent with a paper that sampled paediatric residents who reported that ward rounds are a good opportunity to teach diagnostic investigations, patient management, history taking, physical examination, and time management [4]. In another study by Laskaratos et al., trainees believe that ward rounds are an effective way to educate students on management and diagnostic methods but are ineffective for teaching history-taking, physical examination, building leadership abilities, and learning ethical concepts [1]. A third study by Claridge found that ward rounds provide a good opportunity to learn patient management and diagnostic investigations, according to (91%) of foundation year doctors [3]. According to a fouth paper by Khan et al., (80%) of residents thought that learning about patient management and diagnostic investigation could be done during ward rounds. However,—in contrary to this study and in consistence with Laskaratos et al.—fewer residents (68%) and (62%), thought that ward rounds were an excellent opportunity to learn about gathering histories and performing physical exams. Ward rounds are beneficial for developing communication skills, time management abilities, and record-keeping, was generally agreed upon across studies. The least fruitful learning experience in ward rounds was deemed to be learning basic sciences [4].

The majority of participants in the survey (92.8%) thought that ward rounds might be improved as a learning opportunity. This collective opinion was common throughout the literatute. In a paper by Khan et al. [4], most participants agreed that ward rounds could be revised and rebuilt to create a better learning experience. In another study by Noorani et al. residents reported that "The ward rounds can achieve its full learning potential if planned and organized well, but can become a missed opportunity if the learning environment is unfriendly" [6]. This discrepancy between current quality of ward rounds, and a desired better quality was also reported by paediatric residency trainees in a paper by Grey et al. [7]. This information comes consistent with other findings from the literature such as that of Collet et al., where – as reported- students valued the great learning opportunity ward-rounds are, but felt that greatly depended on the team to which they were attached [8].

Regarding barriers to effective learning on ward rounds, the lack of privacy (77%) and noise in the ward environment (70%) were the most common reported problems faced on ward rounds. More than half of the participants identified lack of nurses (65%), a high patient volume in the wards (63.5%), changes in the team structure (62.5%), a lack of time (58.6%), and patient complaints (58.4%) as obstacles to learning. Fewer participants were bothered by the patients' lunchtimes (42.5%), availability (45.7%), and lack of familiarity (376%) with the patients as barriers, as was the case in the study by laskaratos [1]. Table 3 provides a summary of the challenges encountered during ward rounds.

A systematic review by Khalaf et al. reported barriers to effective learning during ward rounds from 7 studies. As in our study, the barriers included lack of time, interruptions due to the environment, and hierarchies. Other barriers reported in the systematic review included workloads, unarranged schedules, the service-oriented nature of the ward rounds, lack of feedback, and the lack of opportunities to ask questions and be engaged in patient management [9]. As for literature from primary research, lack of time was one of the most reported problems in the literature [1, 3, 4, 6]. Another frequently mentioned factor was the enormous number of patients in wards [1, 3, 4, 6]. Other elements included interruptions frequently [1, 4], afternoon timing, seniors' lack of enthusiasm, team structure, poor time management, and tardy attendance [3].

When asked about what qualities make a good ward round teacher, the participants largely concurred on being enthusiastic about teaching (95.1%), communicating effectively with patients (94.7%), comprehending students' learning needs (94.4%), communicating effectively with them (93.4%), giving feedback (93.1%), and being approachable (91.6%). The majority of participants also agreed on several other points, including teaching slowly and steadily with interest (85%), taking the time to explain things (85.2%), teaching in front of registrars (87.4%), being respected by the students (82.4%), picking engaging topics (79.6%), and having experience instructing medical students (71.4%). Fewer doctors agreed upon characters such as to teach in front of plenty of registrars, stay away from aggressive behaviors and get to know the students (69.5%, 67.9%, and 56.2%, respectively). Table 4 provides a summary of the doctors' opinions regarding the qualities that make a competent ward round teacher.

Laskartos et al. obtained results that are similar to our findings in the literature. Increased time spent on educating, as in spending more time with each patient and having consultants explain the reasoning behind decisions, was a recurring trait [1]. Participants in a study by Khan et al. described a good teacher as enthusiastic towards teaching, provides feedback to students, does not rush, communicates well, and is at consultant level [4]. Teaching by example (such as having a good bedside manner), sharing the attending's thought processes, being approachable and not intimidating, respect for all team members, organized, efficient, and timely round management, and outlining expectations for residents and students were all qualities mentioned by medical professionals in Brita Roy et al.'s study as qualities of a good ward round teacher [2]. For medical students, "Sharing of attending's thought processes" was the most crucial quality, while for faculty, "Be approachable—not intimidating" was the most crucial quality. These qualities are exactly what the Liaison Committee on Medical Education (LCME), which oversees the accreditation of medical universities in the US and Canada, has established as requirements for medical educators. The organization in charge of accrediting medical schools and overseeing the training in foundation programs in the United Kingdom places a strong emphasis on evaluating medical teachers (residents, faculty members, and postdoctoral fellows) using the same standards [10, 11].

Physicians teaching the ward rounds were also questioned about their opinions on what constitutes a good ward-round learner, in the final section of the questionnaire. Virtually all participants believed that it is important to communicate well with the teacher (94.5%), that learning is interesting (94.3%), and that it is important to communicate effectively with patients (93.9%). The majority concurred that having solid information (87.9%) and not rushing was preferable. Also, about half of the doctors (53%) agreed that the students knew them personally was an important factor. Details in Table 5

In the literature, a critical element of effective ward rounds for students was the ability to grasp how experienced physicians arrive at their conclusions [1]. Moreover, having a good bedside manner and being personable were significant qualities. Also, "Sharing the attending's cognitive processes" was highly valued [2]. Further, according to additional research, a teacher must be personable, create rapport among the team members, allow for inquiries, provide high-quality feedback, and be on time and well-prepared, particularly regarding timing. Also, they must be aware of their student's learning requirements and adapt their design iterations. Students mentioned that their teachers' enthusiasm for the subject matter, their willingness to take their time and not rush through the lesson, their ability to communicate with the trainees, and their capacity to offer feedback, while residents said that a teacher's ability to foster discussion in a non-judgmental manner during ward rounds was crucial to learning [4]. From the point of view of the teachers, the most typical qualities of good students in ward rounds were "asks questions" and "fosters discussion", "exhibits commitment", and "takes initiative" [3].

While it is the job of the teacher and the students to design an effective ward round, the goal of this area's investigation was to reflect the perspectives of both parties. It mostly has to do with how juniors and seniors are now positioned on the ward rounds, how they are organized, and if there is enough time available for instruction either during the round or in pre- or post-ward round sessions. Most trainees stated that successful ward rounds have a variety of critical traits, including a supportive learning environment, clinical teaching, effective teaching techniques, articulating expectations, and team management. It is worthy of mention as well that there were multiple papers that supported the use of ward-round tools to create a structured approach to teaching [6, 12].

In overview, this study has many strong points, as it is the first study to be based on such a large sample size that covers an area of wide geographic distribution with a very comprehensive questionnaires—in comparison to other questionnaires in the literature -. It also assesses the perceptions of both the teacher and the student. It aids in identifying the underlying causes of issues that might be encountered when conducting a ward round, most of which can be resolved by following basic organizational principles like ward round structure, or by utilizing professionalism's most essential virtue, time management. However, its drawback was that its questionnaire was distributed electronically. Secondly, because the study relied on a survey-based methodology, there may have been some response bias because participants who were interested in the topic were more likely to complete the questionnaire truthfully. The study makes no comments about consultants' participation.

The results of other studies that were conducted internationally generally agree with the results of this study, which supports the conclusions made in this study and other studies; and confirms that the quality of ward-round teaching is a global issue that is being addressed with nearly identical key controlling factors.

## Conclusion

House officers, medical officers, and registrars of Medicine, Paediatrics, surgery, and Obstetrics and Gynaecology from 50 hospitals around Sudan reported that the overall number of wards rounds they attend per week was 3.1 ± 6.8, which is higher than the average rounds per week reported in the literature. The large majority believed that ward rounds are highly suitable for teaching patient management, and diagnostic investigations, and just suitable to teach history taking, physical examination, communication skills, time management skills, and record keeping. A few agreed that ward rounds are suitable for teaching basic sciences.

Participants agreed that ward rounds could be made into a better learning experience, but obstacles to good ward rounds were: "lack of privacy" and "noise in the ward environment". Other obstacles included" lack of nurses", "a large number of patients in wards,” and others. Most important characteristics for a good teacher ward rounds were “being interested in teaching”,” good communication skills with the patients”,” understanding the learning needs of the students”, and “providing feedback". Ward rounds teachers agreed that good ward-round students "communicate appropriately with the teacher", "are interested in learning", and "communicate appropriately with the patients".

Ward rounds continue to be a powerful educational tool that requires the collaboration of healthcare professionals to protect and prioritize its quantity, quality, and patient experience. Future research should include new educational programs to improve teaching in ward rounds and assess the impact of these new interventions.

## Supplementary Information

**Additional file 1.**

## Data Availability

The data set used and/or analyzed during the study are available from the corresponding author on reasonable request.
