# Supplementary material for: The educational value of ward rounds as a learning and teaching opportunity for house officers, medical officers, and registrars in Sudanese hospitals: a multi-center cross-sectional study
Source: BMC Med Educ. 2023 Jun 8;23:426. doi: 10.1186/s12909-023-04404-z (PMC10248958; doi:10.1186/s12909-023-04404-z)
Supplement: Supplementary file 1 — Additional file 1. [file 12909_2023_4404_MOESM1_ESM.docx]

The educational value of ward rounds as learning and teaching opportunity for House officers, Residents, and Registrars

Name of data collector:…………………………………………………………………………

1_Age:………

2_Gender:………

3_Hospital:………

4_Department:……..

5_Position:

House officer( )

Medical ( )

Registrar( )

6-Deparment :………

7-Years from graduation

8-the number of ward rounds conducted per weeks:……..

9-Total hours spent on ward rounds per week:…….

10-total number of houseofficers and medical working with you in the unit:……..

11-the number of registrars working with you in one unit:…….

12-do you agree that’s word rounds could be made into better experience

strongly Agree( ) agree( ) neither agree or disagree( ) disagree( ) strongly disagree( )

1. Opportunities and obstacles to learning(teaching)on ward rounds:

| *Ward round have been a good opportunity to teach(learn): | Strongly agree | Agree | Neither agree or disagree | Disagree | Strongly disagree |
| --- | --- | --- | --- | --- | --- |
| 1-History Taking |  |  |  |  |  |
| 2-Physical examination |  |  |  |  |  |
| 3-Diagnostic investigations |  |  |  |  |  |
| 4-Patient management |  |  |  |  |  |
| 5-Communication skills |  |  |  |  |  |
| 6-Time Management Skills |  |  |  |  |  |
| 7-Record Keeping |  |  |  |  |  |
| 8-Basic Sciences |  |  |  |  |  |

| *Obstacles to learning (teaching) on ward rounds: | Strongly agree | Agree | Neither agree or disagree | Disagree | Strongly disagree |
| --- | --- | --- | --- | --- | --- |
| 1-Lack of time |  |  |  |  |  |
| 2-Number of patients |  |  |  |  |  |
| 3-Team structure changes to often |  |  |  |  |  |
| 4-i don’t know the patients |  |  |  |  |  |
| 5-ward environment was too noisy |  |  |  |  |  |
| 6-ward environment lack privacy |  |  |  |  |  |
| 7-ward environment lack nursing staff |  |  |  |  |  |
| 8-Patient complaint |  |  |  |  |  |
| 9-patient meal time |  |  |  |  |  |
| 10-patient not available |  |  |  |  |  |

B-what makes a good teacher on ward round:

|  | Strongly agree | Agree | Neither agree or disagree | Disagree | Strongly disagree |
| --- | --- | --- | --- | --- | --- |
| *Someone you know* |  |  |  |  |  |
| *Someone you respect* |  |  |  |  |  |
| *Many consultants in round* |  |  |  |  |  |
| *Present of registrars* |  |  |  |  |  |
| *Interest towards teaching* |  |  |  |  |  |
| *Someone who is not intimidating* |  |  |  |  |  |
| *Someone who is not in hurry* |  |  |  |  |  |
| *Someone who can communicate with you* |  |  |  |  |  |
| *Someone who can communicate with the patient* |  |  |  |  |  |
| *Someone who can provide feedback* |  |  |  |  |  |
| *Approachable* |  |  |  |  |  |
| *Someone who take time to explain* |  |  |  |  |  |
| *The Topic he choses* |  |  |  |  |  |
| *Seem to teach more with medical student* |  |  |  |  |  |
| *Slow and steady ward round with interested doctors are best* |  |  |  |  |  |
| *Someone who understand the learning needs* |  |  |  |  |  |

D-what makes a good student on a ward round:

|  | Strongly agree | Agree | Neither agree or disagree | Disagree | Strongly disagree |
| --- | --- | --- | --- | --- | --- |
| 1-Someone you know |  |  |  |  |  |
| 2-Interest towards learning |  |  |  |  |  |
| 3-good level of knowledge |  |  |  |  |  |
| 4-Someone who is not on hurry |  |  |  |  |  |
| 5-someone who can communicate with you |  |  |  |  |  |
| 6-someone who can communicate with patient |  |  |  |  |  |
